# Professor Virchow on Acclimatisation

**Published:** 1886-10

**Authors:** 


### Professor Virchow on Acclimatisation.

At a recent congress of German naturalists and physicians—a scientific body resembling our British Association, but of wider scope, since it includes medicine—held at Strasburg, Professor Virchow gave an address upon Acclimatisation. He said that he would not discuss the political question as to the propriety of Germany forming colonies, but would point out how wide was the scope for scientific investigation into the subject, an inquiry which might influence State measures. He drew attention to the Amsterdam Congress of Colonial Medicine last year as evidence of the growing desire to learn more concerning the conditions of life and the changes experienced in the human organism under altered climatic influences. When an individual goes to a very different climate he is mostly at first uncomfortable, and after some weeks finds his organism regain its equilibrium; he has accommodated himself to new conditions; his organs have endured material change; he suffers from climatic indisposition or disease. Although the French and English have published much upon the subject, yet researches upon the special changes that precede the onset of disease are entirely wanting. On the other hand the clinical study of tropical disease has been very much advanced. Whoever studies acclimatisation must do so with a view to establish certain geographical limits of ethnological provinces analogous to botanical and zoological provinces. The Professor next proceeded to inquire how far the white races were known to be capable of acclimatisation, and showed that the Semitic were much more capable of it than the Aryan races; and amongst Aryans, the southerners (Portuguese, Spanish, Maltese, etc.) were more so than the northerners. Mixed races with strong Semitic admixture are more easily acclimatised than the pure Aryan stock. These races have developed in North America, the French in Canada, the Yankees in the United States, the English in Australia, but not in its northern, hotter parts. All these inhabit latitudes not far removed from the European, and yet they often become sterile, and the population decreases. Individuals born in the new country of the immigrant population never exceed three generations—for example, the English in India; and in spite of attempts to consolidate the Aryan colonisation of the Indies by means of sanitary measures, it does not succeed. Medical officers of the navy and merchant service, and others, should study these conditions in the light of physiology. What is it that is most at fault? Is tropical anæmia a diminished formation or an increased destruction of blood? Tropical anæmia is found not only in regions where malaria, pernicious relapsing fever, dysentery, or yellow fever prevails, but it occurs apart from these diseases. This increase in blood disintegration excites a great tendency to diseases of the liver, and the liver is the main point of attack for diseases of acclimatisation. To investigate the particular kind of disorders induced by acclimatisation in individual organs is, Professor Virchow said, a paramount duty imposed upon German science. It does not depend upon special examples, but on the scientific determination of the conditions of existence for the vulnerable race of emigrants.

